# Bayesian Geostatistical Modeling of Leishmaniasis Incidence in Brazil

**DOI:** 10.1371/journal.pntd.0002213

**Published:** 2013-05-09

**Authors:** Dimitrios-Alexios Karagiannis-Voules, Ronaldo G. C. Scholte, Luiz H. Guimarães, Jürg Utzinger, Penelope Vounatsou

**Affiliations:** 1 Department of Epidemiology and Public Health, Swiss Tropical and Public Health Institute, Basel, Switzerland; 2 University of Basel, Basel, Switzerland; 3 Centro de Pesquisas René Rachou, Fiocruz, Belo Horizonte, Brazil; 4 Serviço de Imunologia, Complexo Hospitalar Universitário Prof. Edgard Santos, Universidade Federal da Bahia, Bahia, Brazil; RTI International, United States of America

## Abstract

**Background:**

Leishmaniasis is endemic in 98 countries with an estimated 350 million people at risk and approximately 2 million cases annually. Brazil is one of the most severely affected countries.

**Methodology:**

We applied Bayesian geostatistical negative binomial models to analyze reported incidence data of cutaneous and visceral leishmaniasis in Brazil covering a 10-year period (2001–2010). Particular emphasis was placed on spatial and temporal patterns. The models were fitted using integrated nested Laplace approximations to perform fast approximate Bayesian inference. Bayesian variable selection was employed to determine the most important climatic, environmental, and socioeconomic predictors of cutaneous and visceral leishmaniasis.

**Principal Findings:**

For both types of leishmaniasis, precipitation and socioeconomic proxies were identified as important risk factors. The predicted number of cases in 2010 were 30,189 (standard deviation [SD]: 7,676) for cutaneous leishmaniasis and 4,889 (SD: 288) for visceral leishmaniasis. Our risk maps predicted the highest numbers of infected people in the states of Minas Gerais and Pará for visceral and cutaneous leishmaniasis, respectively.

**Conclusions/Significance:**

Our spatially explicit, high-resolution incidence maps identified priority areas where leishmaniasis control efforts should be targeted with the ultimate goal to reduce disease incidence.

## Author Summary

Leishmaniasis is a neglected tropical disease that causes approximately 20 to 40 thousand deaths every year. In Brazil, more than 600,000 clinical cases of leishmaniasis have been reported since 1990. Almost 90% of these cases are due to cutaneous leishmaniasis, whereas the remaining 10% are due to visceral leishmaniasis. Understanding of disease transmission, together with model-based incidence maps, will assist in designing and optimizing control efforts. We used reported leishmaniasis incidence data in Brazil covering the period between 2001 and 2010 to explore the association of the disease with climatic, environmental, and socioeconomic variables, and to predict its spatial distribution using Bayesian geostatistical models. We produced countrywide high spatial resolution maps for both forms of leishmaniasis and estimated the number of infected people, stratified by state. We believe that our incidence maps are useful to prioritize the spatial targeting of prevention and control.

## Introduction

Leishmaniasis is a group of neglected tropical diseases that are caused by parasites of the genus *Leishmania*. The parasites are transmitted by female phlebotomine sandflies and the disease occurs in human in two different clinical forms: (i) cutaneous (CL, referring to the greater group of American tegumentary leishmaniasis), which causes skin or mucosal lesion; and (ii) visceral (VL), which affects organs such as the liver and spleen [1]. The latter, if not diagnosed and treated in the early stages, is usually fatal [2], [3].

In 2002, the World Health Organization (WHO) estimated that 350 million people were at risk of leishmaniasis, with approximately 2 million (1.5 million CL and 0.5 million VL) cases and 59,000 deaths [4]. Recently, 98 countries reported endemic transmission, with an estimated 0.7–1.2 and 0.2–0.4 million new cases per year for CL and VL, respectively. Deaths due to VL are estimated between 20,000 and 40,000 [5]. The burden of leishmaniasis has been increasing worldwide [2], [6]. In Brazil, for example, the number of CL cases climbed from 6,335 in 1984 to 30,030 in 1996 [7]. From 1990 to 2007 some 560,000 new cases of leishmaniasis were reported, primarily CL [3], [8]. However, after 2005, the total number of CL cases has dropped and remained stable, just above 20,000.

Strategies for the control of leishmaniasis in Brazil have not changed over the past 60 years, which might explain why incidence did not decrease [9]. According to World Health Assembly (WHA) resolution 60.10, put forward in 2007, a well-defined implementation of a control program for leishmaniasis is still lacking [10]. The difficulties in case reporting and detection are the main obstacles for such a program. At the same time, due to heterogeneity between the sandfly species, vector control introduces high costs. Effective control requires reliable maps of the spatial distribution of the disease, as well as the number of affected people, so that treatment and other control interventions can be implemented most cost-effectively.

Bayesian geostatistical models have been applied in the mapping of malaria [11]–[14] and neglected tropical diseases [15]–[18]. Geostatistical models relate the disease data with potential predictors and quantify spatial dependence via the covariance matrix of a Gaussian process facilitated by adding random effects at the observed locations. However, covariance matrix computations hamper implementation of the models on data collected over large number of locations (>1,000). Different methodologies have been proposed to address this issue (for a recent review see [19]). A predictive process approach, developed by Banerjee et al. (2008) [20], has been successfully applied in infectious disease mapping (see, for example, [18]). Lindgren et al. (2011) [21] showed that Gaussian Markov random fields [22] can be used in geostatistical settings. Rue and colleagues (2009) [23] provide fast computational algorithms for latent Gaussian models, based on integrated nested Laplace approximations (INLA).

There are only few studies that assessed the spatio-temporal distribution, including underlying risk factors, of leishmaniasis. Chaves et Pascual (2006) [24] explored the temporal association of CL cases in Costa Rica by taking into account climatic variables. Chaves et al. (2008) [25] used negative binomial models with breakpoints to analyze CL incidence in Costa Rica. Valderrama-Ardila et al. (2010) [26] studied environmental determinants of CL incidence in an area of Colombia, using spatial models. In Colombia, the probability of CL presence based on ecological zones and environmental variables was explored by King et al. (2004) [27]. In Argentina, Salomón et al. (2012) [28] modeled CL incidence using maximum entropy modeling. To date, efforts for estimating the associated risk and the predicted spatial distribution of leishmaniasis in Brazil are limited to small geographical areas. For instance, Shimabukuro et al. (2010) [29] analyzed CL transmission in the state of São Paulo by using data on sandfly species presence, while Machado-Coelho et al. (1999) [30] investigated spatio-temporal clustering in south-east Brazil. Jirmanus et al. (2012) [31] examined seasonal variation of CL incidence in Corte de Pedra over a 20-year period and analyzed demographic characteristics of CL patients. Werneck and Maguire (2002) [32] used spatial models, with one socioeconomic and one environmental covariate to explore VL incidence in the city of Teresina. Assunção et al. (2001) [33] predicted VL rates in Belo Horizonte employing spatio-temporal models without including climatic or socioeconomic covariates. The Ministry of Health (MoH) in Brazil has reported incidence maps for the whole country but without the use of predictors and of Bayesian geostatistical approaches [34], [35]. More recently, Alvar et al. (2012) [5] provided worldwide estimates of leishmaniasis and included incidence maps of Brazil corresponding to raw data aggregated by state.

In this study, we analyzed incidence data of CL and VL obtained by the information system for notifiable diseases (ISND) during 2001 to 2010 from the MoH in Brazil. We employed Bayesian geostastical negative binomial models, fitted via INLA to predict the incidence of the diseases, using climatic, environmental, and socioeconomic covariates. We produced countrywide high resolution maps for leishmaniasis and estimated the number of infected people at the unit of the state. The generated incidence maps and estimates might be useful for decision-makers to prioritize intervention areas, and optimizing resources allocation to render control and elimination efforts most cost-effective.

## Methods

### Ethics Statement

We report a geospatial analysis of CL and VL incidence data in Brazil. The data were readily obtained from existing databases. Hence, there are no specific ethical considerations.

### Leishmaniasis Incidence Data

**Figure 1:**
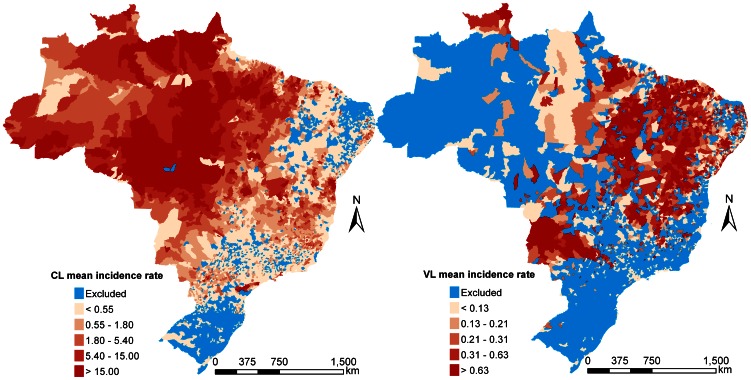
Raw incidence rates. Raw incidence rates (per 10,000) averaged over a 10-year period (2001–2010) for cutaneous leishmaniasis (left) and visceral leishmaniasis (right). Municipalities colored in blue, were excluded from analysis due to missing data.

Annual incidence data extracted from ISND, were obtained from 3,895 (for CL) and 2,176 (for VL) municipalities of Brazil. We have considered autochthonous cases. The municipalities chosen for the analysis were the ones with reported cases (including zeros) for at least one year between 2001 and 2010. Figure 1 shows the municipalities with incidence data and the 10-year mean incidence rate for both CL and VL.

### Climatic and Environmental Data

Climatic data, including altitude, were extracted from Worldclim Global Climate Data [36]. These data consist of 19 bioclimatic variables. Environmental data were obtained from MODIS [37]. Land surface temperature (LST) data were used as proxies of day and night temperature. The normalized difference vegetation index (NDVI) and enhanced vegetation index (EVI) were considered as proxies for moisture and vegetation. Details of the data sources are summarized in Table 1. Municipality level estimates were obtained in ArcMap [38] by aggregating the high resolution data.

**Table 1:** Climatic and environmental predictors used for geostatistical modeling of leishmaniasis in Brazil.

Source	Data type	Data period	Temporal resolution	Spatial resolution
Shuttle Radar Topography Mission (SRTM) data	Digital elevation model (DEM)	2000	Once	1 km
Moderate Resolution Imaging Spectroradiometer (MODIS)/Terra	Land surface temperature (LST) for day and night	2005–2009	8 days	1 km
	Normalized difference vegetation index (NDVI)	2005–2009	16 days	1 km
	Enhanced vegetation index (EVI)	2005–2009	16 days	1 km
Worldclim global climate	Annual mean temperature	1950–2000	Once	1 km
	Mean temperature diurnal range	1950–2000	Once	1 km
	Isothermality	1950–2000	Once	1 km
	Temperature seasonality	1950–2000	Once	1 km
	Maximum temperature of warmest month	1950–2000	Once	1 km
	Maximum temperature of coldest month	1950–2000	Once	1 km
	Temperature annual range	1950–2000	Once	1 km
	Mean temperature of wettest quarter	1950–2000	Once	1 km
	Mean temperature of driest quarter	1950–2000	Once	1 km
	Mean temperature of warmest quarter	1950–2000	Once	1 km
	Mean temperature of coldest quarter	1950–2000	Once	1 km
	Annual precipitation	1950–2000	Once	1 km
	Precipitation of wettest month	1950–2000	Once	1 km
	Precipitation of driest month	1950–2000	Once	1 km
	Precipitation seasonality	1950–2000	Once	1 km
	Precipitation of wettest quarter	1950–2000	Once	1 km
	Precipitation of driest quarter	1950–2000	Once	1 km
	Precipitation of warmest quarter	1950–2000	Once	1 km
	Precipitation of coldest quarter	1950–2000	Once	1 km

### Socioeconomic Data

The socioeconomic indicators used in our study are summarized in Table 2. They include: (i) rural population and human development index (HDI) for the year 2000 provided by the *Instituto Brasileiro de Geografia e Estatística* (IBGE); (ii) unsatisfied basic needs (UBN) for 2000 provided by the Pan American Health Organization (PAHO/WHO); and (iii) infant mortality rate (IMR) for 2000 and human influence index (HII) for 2005 obtained by the Center for International Earth Science Information Network (CIESIN) [39], [40]. Population data for 2010 at municipality level were available from IBGE, while population density at a spatial resolution of 5
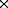
5 km was obtained from CIESIN [41].

**Table 2:** Socioeconomic predictors used for geostatistical modeling of leishmaniasis in Brazil for 2001–2010.

Source	Data type	Data period	Resolution
Instituto Brasileiro de Geografiae Estatística (IBGE) (census data)	Population data	2010	Municipality
	Human development index (HDI)	2000	Municipality
	Rural population	2000	Municipality
Pan American Health Organization (unsatisfied basic needs) (census data)	Bras0_3 (% of pupils enrolled in primary school)	2000	Municipality
	Bras0_4 (% of pupils completing primary school)	2000	Municipality
	Bras0_5 (rate literacy 15 to 24 years)	2000	Municipality
	Bras0_6 (girls and boys primary school)	2000	Municipality
	Bras0_7 (girls and boys high school)	2000	Municipality
	Bras0_8 (girls and boys undergraduate school)	2000	Municipality
	Bras0_9 (relation literacy women and men 15 to 24 years)	2000	Municipality
	Bras0_10 (% women with non-farming occupation)	2000	Municipality
	Bras0_11 (% people with potable water at home)	2000	Municipality
	Bras0_12 (% people with sanitation at home)	2000	Municipality
	Bras0_13 (% people with energy at home)	2000	Municipality
	Bras0_14 (% people that own their house)	2000	Municipality
	Bras0_15 (index secure tenure house)	2000	Municipality
	Bras0_16 (unemployment rate)	2000	Municipality
	Bras0_17 (% of houses with phone)	2000	Municipality
	Bras0_18 (% of house with computer)	2000	Municipality
	Bras2_11 (% of people overcrowding)	2000	Municipality
	Bras2_15 (% of people subsistence)	2000	Municipality
	Infant mortality rate (IMR)	2000	Municipality
Center for International Earth Science Information Network (CIESIN)	Human influence index (HII)	2005	1 km

### Statistical Analysis

The incidence data were modeled via negative binomial regression. Exploratory analysis was carried out in R [42] to assess linearity of the covariates. For continuous covariates, we constructed three new categorical variables with 2, 3, and 4 categories, based on the quantiles of the variables' distribution. The Akaike's information criterion (AIC) was used to select between a categorical or a linear form of each variable. To quantify the temporal trend, we included a binary variable, splitting the 10-year period in two phases, 2001–2005 and 2006–2010.

Gibbs variable selection [43] was performed in WinBUGS [44] with the inclusion of an independent random effect at municipality level and a year specific auto-correlated term. All the covariates were assigned a 0.5 prior probability to be included in the final model. The total number of candidate covariates was 45.

The covariates giving rise to the model with the highest posterior probability were subsequently used to fit a Bayesian geostatistical negative binomial model with spatially structured random effects at municipality level. The spatial correlation was considered to be decreasing with distance between any pair of locations. The temporal random effects were modeled by auto-regressive terms of order 1. More specifically, we assumed that the reported number of CL and VL cases, for location 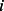 and year 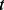, follow a negative binomial distribution with mean 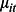 and dispersion parameter 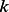. Covariates and random effects were modeled on the 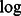 scale of 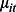, that is 
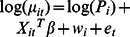
, where 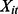 and 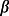 are the vectors of covariates and coefficients, respectively, and 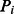 is the population of the 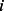-th municipality. The spatial random effects 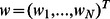 take into account the spatial dependence of the data by assuming they follow a zero-mean multivariate normal distribution with Matérn covariance function (see, for example, [45]). 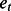 is the auto-correlated error term with 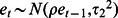 for 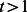, and 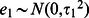 with 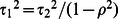, and 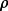 is the auto-correlation.

The large number of municipalities included in our modeling approach challenges geostatistical model fit, and thus resulting in extremely slow Markov chain Monte Carlo (MCMC) runs. To overcome computational burden, we estimated model parameters via INLA, using the homonymous R-package (available at www.r-inla.org). Details on model fit and the related R-code are provided in the supporting information texts S1 and S2, respectively.

Model validation was performed by fitting the model to a randomly selected subset of 80% of the locations and predicting the mean of the remaining 20% (test data). Bayesian credible intervals (BCI) of 95% probability are calculated and the percentage of observations included in these intervals is reported (coverage), as well as the square root of the mean square error (RMSE) of the test data.

A number of municipalities had not reported any cases of leishmaniasis for some years. As it was unclear whether these missing values in our dataset corresponded to true zeros or a lack of reporting cases, a separate analysis was carried out with missing values considered as zeros.

## Results

### Descriptive Results

**Figure 2:**
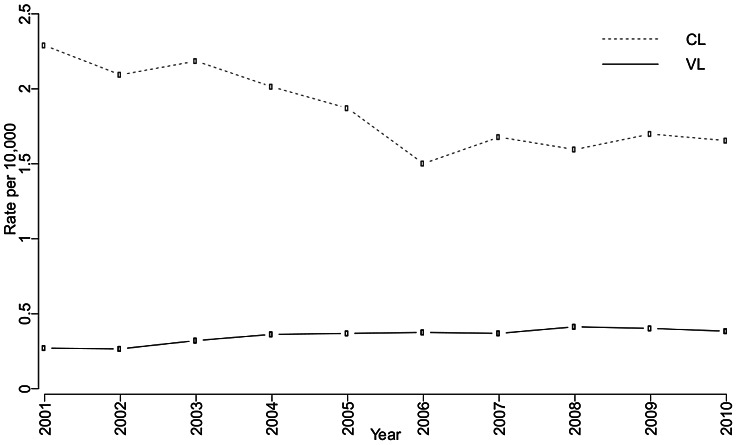
Temporal trend of observed countrywide incidence rates per 10,000.

Figure 2 shows the annual incidence rates of CL and VL per 10,000 people in Brazil for the period 2001–2010. A decrease of CL rates is observed after 2005, while VL rates remained stable. The maximum annual number of cases at the unit of the municipality was 1,820 for CL (Manaus) and 262 for VL (Araguaína).

### Model Estimates

Estimates, BCIs, and confidence intervals (CIs) of the multivariate Bayesian geostatistical and non-spatial models for CL are presented in Table 3. After 2005, the incidence of CL dropped by approximately 20%, which is in line with the results shown in Figure 2. Higher temperature diurnal range, temperature of wettest quarter, annual precipitation, precipitation seasonality, precipitation of warmest quarter, and EVI are positively associated with CL. On the other hand, higher LST is negatively associated with CL incidence. The following socioeconomic variables were associated with low incidence rates of CL: percentage of people with potable water at home, percentage of people with sanitation, percentage of people that own their house, and HII. A higher incidence rate was observed for men, as revealed by the negative relation between the CL incidence and the percentage of women living in an area.

**Table 3:** Parameter estimates for cutaneous leishmaniasis (CL) in Brazil for 2001–2010.

	Bayesian geostatistical	Non-spatial
Variable	IRR (95% BCI)	IRR (95% CI)
**Mean temperature diurnal range (°C)**		
<9.36	1.00	1.00
9.36–10.90	1.46 (1.19, 1.78)	1.00 (0.94, 1.06)
10.90–11.86	1.79 (1.42, 2.27)	1.15 (1.08, 1.22)
>11.86	2.08 (1.56, 2.75)	1.62 (1.50, 1.75)
**Mean temperature of wettest quarter (°C)**	1.30 (1.18, 1.44)	1.19 (1.16, 1.22)
**Annual precipitation (mm)**	1.70 (1.54, 1.88)	1.24 (1.21, 1.27)
**Precipitation seasonality**	1.71 (1.50, 1.95)	1.13 (1.10, 1.16)
**Precipitation of warmest quarter (mm)**		
<207	1.00	1.00
207–369	1.20 (0.99, 1.44)	1.18 (1.12, 1.25)
369–530	1.29 (1.54, 1.88)	1.67 (1.55, 1.81)
>530	0.88 (0.66, 1.15)	0.74 (0.68, 0.81)
**EVI**		
<35.78	1.00	1.00
35.78–39.06	1.31 (1.18, 1.46)	1.70 (1.61, 1.79)
39.06–42.73	1.65 (1.45, 1.89)	1.82 (1.71, 1.93)
>42.73	2.14 (1.46, 2.54)	2.39 (2.22, 2.57)
**Day LST (°C)**	0.74 (0.66, 0.83)	0.81 (0.78, 0.83)
**% People with potable water at home**		
<40.57	1.00	1.00
40.57–71.72	1.00 (0.90, 1.12)	1.18 (1.12, 1.25)
71.72–95.69	0.78 (0.67, 0.92)	0.56 (0.52, 0.60)
>95.69	0.68 (0.56, 0.84)	0.39 (0.36, 0.43)
**% People with sanitation at home**	0.81 (0.76, 0.86)	0.82 (0.79, 0.84)
**% People that own their house**	0.92 (0.88, 0.96)	0.90 (0.88, 0.92)
**% of women**	0.82 (0.77, 0.86)	0.74 (0.72, 0.76)
**HII**		
<17.02	1.00	1.00
17.02–20.30	0.86 (0.76, 0.98)	0.79 (0.75, 0.84)
20.30–23.48	0.73 (0.63, 0.85)	0.54 (0.50, 0.57)
>23.48	0.70 (0.59, 0.83)	0.45 (0.42, 0.48)
**Period**		
2001–2005	1.00	1.00
2005–2010	0.80 (0.67, 0.95)	0.83 (0.80, 0.86)
	**Mean (95% BCI)**	
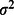 **(spatial variance)**	1.45 (1.35, 1.56)	
**Range (km)**	88.3 (82.2, 94.9)	
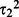 **(temporal variance)**	0.02 (0.01, 0.03)	
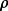 **(temporal correlation)**	0.74 (0.30, 0.95)	
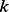 **(dispersion)**	2.23 (2.15, 2.32)	

Parameter estimates for CL based on a Bayesian geostatistical and a multiple non-spatial negative binomial regression model. Coefficients are presented in terms of incidence rate ratios (IRR). BCI, Bayesian credible interval; CI, confidence interval.

Parameter estimates of VL are summarized in Table 4. The most suitable climatic and environmental factors for VL are: low altitude, low annual precipitation, increased temperature diurnal range, and none extreme precipitation during the warmest quarter. With regard to socioeconomic variables, similar as in CL, effects of the two socioeconomic variables (i.e., percentage of people with sanitation at home and percentage of people that own their house) were associated with lower incidence of VL. Mean temperature diurnal range was the only climatic variable associated with a lower rate of VL incidence.

**Table 4:** Parameter estimates for visceral leishmaniasis (VL) in Brazil for 2001–2010.

Variable	Bayesian geostatistical IRR (95% BCI)	Non-spatial IRR (95% CI)
**Altitude (m)**		
<163	1.00	1.00
163–341	0.93 (0.75, 1.16)	0.76 (0.70, 0.84)
341–560	0.96 (0.74, 1.25)	0.70 (0.63, 0.78)
>560	0.81 (0.61, 1.09)	0.53 (0.48, 0.60)
**Mean temperature diurnal range (°C)**		
<9.00	1.00	1.00
9.00–10.38	1.17 (0.92, 1.48)	1.58 (1.45, 1.73)
10.38–11.80	1.81 (1.33, 2.47)	3.05 (2.79, 3.34)
>11.80	2.47 (1.74, 3.48)	4.70 (4.26, 5.20)
**Annual precipitation (mm)**		
<832	1.00	1.00
832–1212	0.89 (0.73, 1.10)	0.81 (0.74, 0.88)
1212–1512	0.64 (0.48, 0.85)	0.63 (0.57, 0.69)
>1512	0.59 (0.42, 0.82)	0.59 (0.52, 0.65)
**Precipitation of warmest quarter (mm)**		
<130	1.00	1.00
130–205	1.10 (0.89, 1.37)	1.25 (1.15, 1.36)
205–359	0.88 (0.67, 1.15)	1.11 (1.02, 1.21)
>359	0.54 (0.39, 0.76)	0.68 (0.60, 0.76)
**Precipitation of coldest quarter (mm)**	1.12 (0.97, 1.29)	1.26 (1.20, 1.31)
**% People with sanitation at house**		
<2	1.00	1.00
2−25	0.91 (0.82, 1.02)	0.89 (0.83, 0.96)
>25	0.62 (0.54, 0.73)	0.60 (0.55, 0.66)
**% People that own their house**		
<81.51	1.00	1.00
81.51–87.23	0.88 (0.77, 1.00)	1.04 (0.96, 1.13)
87.23–90.76	0.88 (0.77, 1.01)	0.99 (0.91, 1.07)
>90.76	0.71 (0.61, 0.83)	0.86 (0.79, 0.94)
**Period**		
2001–2005	1.00	1.00
2006–2010	1.16 (0.94, 1.35)	1.24 (1.18, 1.31)
	**Mean (95% BCI)**	
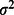 **(spatial variance)**	1.09 (0.97, 1.23)	
**Range (km)**	109.1 (96.3, 124.6)	
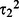 **(temporal variance)**	0.01 (0.00, 0.03)	
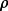 **(temporal correlation)**	0.35 (−0.25, 0.86)	
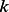 **(dispersion)**	1.74 (1.62, 1.88)	

Parameter estimates for VL based on a Bayesian geostatistical and a multiple non-spatial negative binomial regression model. Coefficients are presented in terms of incidence rate ratios (IRR). BCI, Bayesian credible interval; CI, confidence interval.

For both diseases the spatial variance was higher than the temporal one. Estimates of the range parameter indicate that spatial correlation becomes negligible for distances above 88.3 and 109.1 km for CL and VL, respectively.

### Model Validation

The model of CL had a RMSE of 14.2 when predicted over the 20% randomly selected locations. One third of the cases (34%) were included in 95% BCIs of the posterior predictive distribution. The respective estimates for VL were 4.11 and 23%.

### Incidence Maps

**Figure 3:**
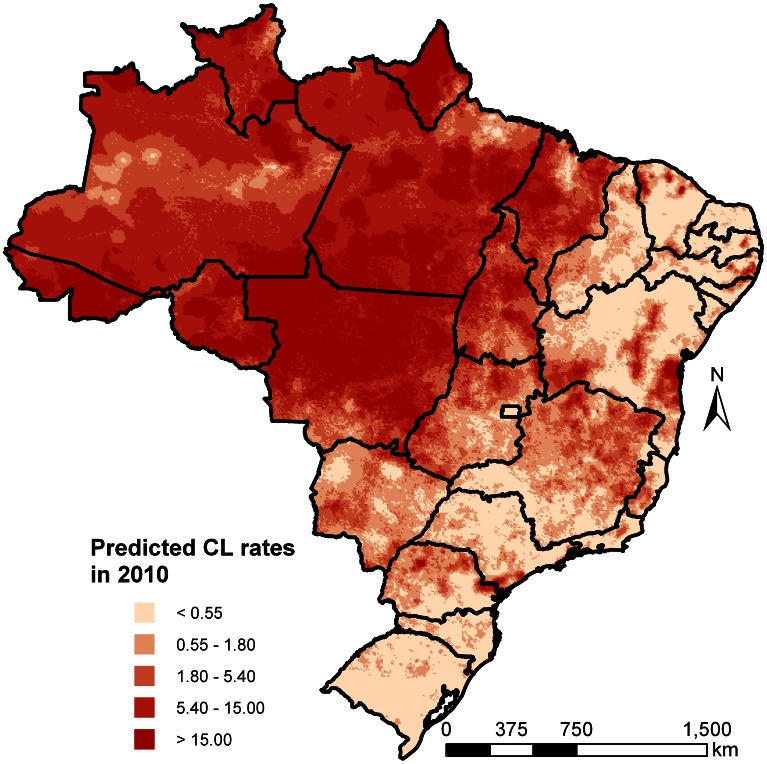
Geostatistical model-based predicted incidence rates per 10,000 for cutaneous leishmaniasis in Brazil in 2010.

**Figure 4:**
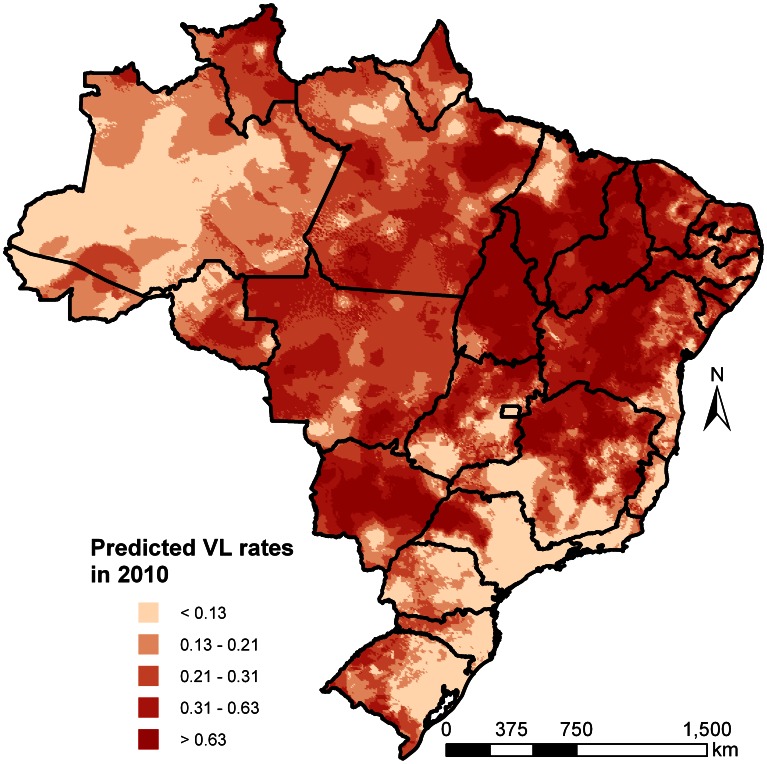
Geostatistical model-based predicted incidence rates per 10,000 for visceral leishmaniasis in Brazil in 2010.

**Figure 5:**
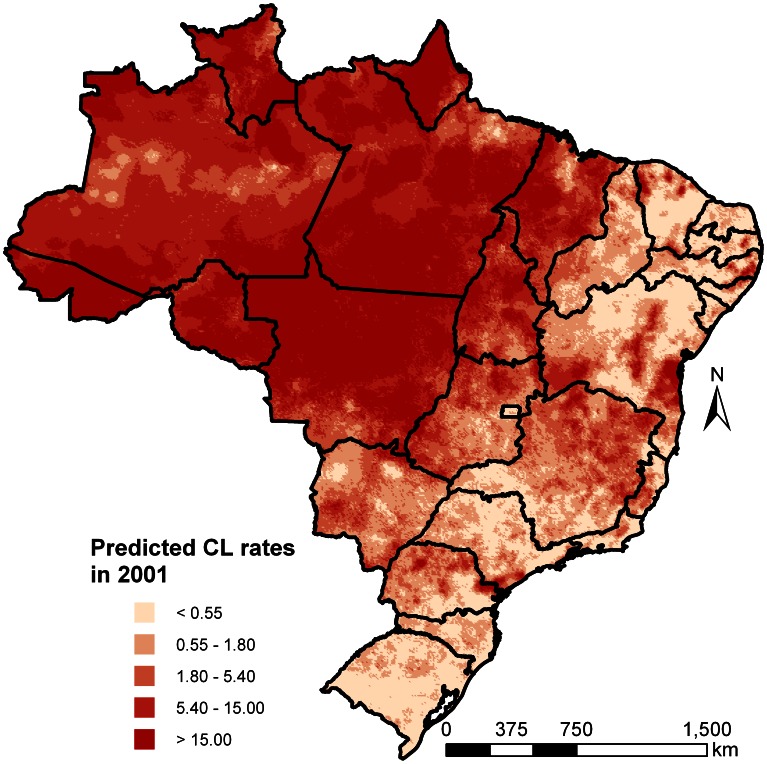
Geostatistical model-based predicted incidence rates per 10,000 for cutaneous leishmaniasis in Brazil in 2001.

Model-based predictions were obtained over a grid of 136,841 pixels at 8
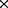
8 km spatial resolution. The rates (per 10,000 people) of the predictions for CL and VL in 2010 are depicted in Figures 3 and 4, respectively. The decreasing trend of CL cases is apparent by comparing the maps for the year of 2010 (Figure 3) with that of 2001 (Figure 5). For instance, in 2010 lower rates were observed in west and north-west Brazil in the states of Amazonas and Roraima. Incidence maps under the assumption that missing cases were zeros are provided in supporting information text S3.

### Country and State Estimates

The incidence rate map was overlaid with the population map of Brazil to estimate the number of cases per pixel. By aggregating the number of pixels per state, we estimated the number of infected people for both diseases (Table 5). The total number of cases predicted for 2010 was 30,189 (standard deviation (SD): 7,676) for CL and 4,889 (SD: 288) for VL. The highest prediction for CL occurred in the state of Pará (4,332), while for VL in Minas Gerais (693). The corresponding country and state estimates under the assumption that missing cases were zeros are reported in supporting information text S3.

**Table 5:** Country and state predicted cases of cutaneous leishmaniasis (CL) and visceral leishmaniasis (VL) in Brazil in 2010.

State	CL cases (SD)	VL cases (SD)
Acre	1,511.0 (647.3)	7.8 (3.0)
Alagoas	151.7 (30.4)	115.4 (20.4)
Amapá	466.5 (52.1)	8.3 (4.9)
Amazonas	1,829.1 (858.0)	26.6 (6.2)
Bahia	3,402.3 (905.0)	467.1 (50.7)
Ceará	1,637.2 (345.0)	599.4 (100.6)
Distrito Federal	67.3 (32.4)	14.1 (5.6)
Espírito Santo	248.6 (75.7)	31.6 (8.0)
Goiás	634.6 (169.0)	89.0 (11.5)
Maranhão	3,417.3 (855.3)	500.0 (59.8)
Mato Grosso	3,383.2 (1461.1)	68.3 (9.9)
Mato Grosso do Sul	258.0 (488.8)	204.1 (65.0)
Minas Gerais	1,947.6 (110.4)	692.7 (67.7)
Pará	4,331.6 (1129.0)	406.6 (52.1)
Paraíba	190.1 (195.7)	79.1 (10.7)
Paraná	1,082.6 (412.6)	82.3 (17.7)
Pernambuco	895.0 (40.2)	184.2 (25.4)
Piauí	199.4 (55.7)	276.0 (40.0)
Rio de Janeiro	281.7 (748.3)	48.0 (16.5)
Rio Grande do Norte	77.3 (17.5)	108.0 (15.2)
Rio Grande do Sul	182.4 (58.8)	109.0 (24.1)
Rondônia	1,896.8 (724.2)	32.1 (9.6)
Roraima	173.8 (171.5)	7.9 (2.6)
Santa Catarina	194.3 (78.8)	61.0 (18.7)
São Paulo	1,006.8 (90.6)	343.4 (28.3)
Sergipe	70.2 (15.7)	68.1 (11.4)
Tocantins	652.9 (229.7)	258.5 (40.1)
Total	30,189.1 (7,675.8)	4,888.7 (288.3)

SD, standard deviation.

## Discussion

We provide countrywide, model-based incidence maps for both cutaneous and visceral leishmaniasis in Brazil, at a high spatial resolution (8
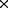
8 km). Furthermore, we explored the underlying spatial processes, identified risk factors, and displayed high incidence areas. Taken together, our investigations provide a deeper understanding of the determinants of the two diseases. We employed Bayesian geostatistical models fitted on readily available incidence data from the MoH in Brazil, and used Bayesian variable selection to identify environmental and socioeconomic predictors. Although analyses for mapping leishmaniasis incidence data at state level were previously conducted, they rarely used rigorous statistical modeling approaches to take into account spatio-temporal correlations. However, ignoring correlation, risk factor analyses and predictions may be incorrect.

Our results indicate that humid warm climates with high vegetation indexes are associated with high incidence of CL. In contrast, high temperatures are associated with lower incidence of CL. A study in sub-Andean zone in Colombia [26] also reported a negative association between incidence of CL and temperatures exceeding a minimum cut-off of 20.6°C. The association between vegetation and CL incidence found in our study, corroborates previous observations [26] and may point to the role of deforestation driving CL outbreaks due to vector proliferations [46]. Our analysis suggests a higher incidence rate for males, which has also been reported by the MoH in Brazil [34]. These observations might be explained by gender-specific occupational exposure within endemic areas [47]. The climatic conditions suitable for VL transmission are different to those of CL. A spatial analysis, done for the Islamic Republic of Iran, including environmental covariates, revealed that precipitation was positively associated with CL incidence [48]. On the other hand, the incidence of VL was not associated with the presence of vegetation and the role of annual precipitation is negative, which might reflect extreme conditions. An inverse relation of VL incidence and the mean of 3-year precipitation has been reported in a previous study in north-east Brazil [49]. VL shows higher incidence rates in lowlands as revealed by the negative altitude effect, which is in accordance with previous observations [50].

There was an association between socioeconomic factors with the diseases' incidence, confirming earlier reports that the population with the lowest socioeconomic status is affected the most [2]. Indeed, the higher the proportion of people with access to clean water and improved sanitation, the lower the infection rate. In fact, control programs which focus on improving sanitation were associated with lower incidence rates. The intimate connection between poor living conditions and leishmaniasis has been discussed before [32].

Our analysis underscores the importance of rigorous geostatistical modeling in identifying factors related to transmission. Results from non-spatial analogue models may identify different predictors or even estimate a different direction of the effects. The strong spatial correlations estimated by our models may suggest that we missed out important spatially structured predictors. For instance, vector and reservoir presence would drive such models. In addition, the analysis was based on incidence data aggregated over municipalities. Since the observed data are already available at municipality level, it is unlikely that predictions at the same level would be more informative. The strength of the predictive models is their ability to generate estimates in areas where no data are available. Data at higher spatial resolution may be able to obtain more precise estimates.

Incidence data were missing for some municipalities and some years in the 10-year observation period. These missing values could indicate true zero cases; however “zeros” have been recorded in the dataset in addition to the missing data. In our analysis we treated non-reported cases as missing. This may partially explain the overestimation of the total number of cases. To address this issue, we carried out a separate analysis, assuming that non-reported cases are zeros. The point estimates of predicted cases per state and the smooth maps are given in the supporting information text S3. This analysis provided estimates of the total numbers of cases in the country which were closer to the reported ones in ISND. Maia-Elkhoury et al. (2007) [51] estimated 42% and 45% (depending on source comparison) of under-reporting for VL in ISND using a capture-recapture method. Alvar et al. (2012) [5] pointed that these percentages correspond to 1.3–1.7-fold degrees of under-reporting. Our total VL predicted cases fall within this interval. We are not aware of similar estimation of CL under-reporting for ISND. By assuming a similar amount of under-reporting for CL (due to the same source), the total number of predicted cases of our analysis lies within the above interval. Overestimation of the predicted cases may also arise because the incidence is very low and models cannot predict exact zeros. An estimate slightly higher than zero at pixel level will overestimate the total number of cases. The more pixels aggregated, the larger the overestimation. Hence, the model will overestimate, for example, treatment needs. Rounding to zero pixel-level cases predicted less than 0.1, the total number of model-based estimates of VL cases at country level drops to 3,320 from 4,889 and for CL to 28,164 from 30,189. However, this cut-off is arbitrary. For decision making, thresholds of predicted cases could be applied. These could be defined by some optimality criteria, which balance cost of not providing timely treatment on one hand and cost of administering drugs which were not required on the other hand.

Our study has several limitations that are offered for consideration. Brazil is the fifth largest country of the world and can be divided into different ecological zones. We assumed a single relation between risk factors and the incidence of leishmaniasis, which might not be able to capture properly the geographical distribution. Non-stationary models allowing for different spatial dependencies and covariate distribution in a specific area [52], [53] may improve predictive ability. We did not include a space-time interaction, but instead assumed a constant spatial process over time. To perform such an analysis, data are needed for specific time periods and for each municipality. In our study, this would require either dropping a large number of municipalities from the study or incorporating the estimation of their values in the modeling process. The latter might result in identifiability problems of the parameters, and hence, we only considered additive effects. We assumed constant effects of the predictors over time and therefore could not explain the temporal trends of CL from the trends of the predictors considered in the study. The coverages of the test data for both diseases might seem low, but do not account for the zero cases. The 2.5% quantile cannot be zero, and thus all the zero incidence cases will be missed. To illustrate this, we rounded the lower quantile (which of course increases the credibility level) and recalculated the coverages resulting in 66% for CL and 71% for VL. In addition, 50% and 38% municipalities had 0 reported cases for CL and VL, respectively. Giardina et al. (2012) [54] showed that zero-inflated (ZI) models gave better predictions than standard geostatistical models for predicting malaria risk using sparse malaria survey data. ZI models with an invariable probability of ZI were also fitted, but according to the deviance information criterion (DIC) they showed similar fits to the data and the probability of ZI was very low (of the magnitude 10^−6^). Cross-validatory measures (i.e., coverage and RMSE) did not improve when ZI models were fitted. Non-linearity was addressed by categorizing the predictors. Alternative approaches (i.e., polynomial terms or splines) may provide more flexible ways to model the relation between disease and predictors, and potentially give more accurate estimates. We have chosen categorical covariates because they offer easier epidemiological interpretation.

In conclusion, we present the first high-resolution model-based estimates of CL and VL in Brazil. We used INLA, a novel inferential approach in the field of neglected tropical diseases. Our incidence maps, together with the predicted number of CL and VL cases, constitute useful tools for decision making and prioritization of disease control intervention. Recent developments in Bayesian geostatistical computation (e.g., INLA) already enable analyses of surveillance data in almost “real” time. Updates of these maps could be automatized, and hence performed shortly after data collection and reporting. We anticipate that in near future surveillance programs will integrate these methods in their systems. The possibility to aggregate over any desired level, such as the catchment area of health facilities, would further help planning drug delivery and other control measures. In particular, these maps could identify communities where enhanced prevention measures are warranted. Environmental predictors are important for identifying high incidence areas, while improving socioeconomic status might constitute the single most important factor to enhance control programs. The current methodology should be further developed to address the aforementioned limitations and provide more accurate spatial and temporal predictions of leishmaniasis incidence.

## Supporting Information

Alternative Language Abstract S1 **Translation of the abstract into Portuguese by R. G. C. Scholte.**
(DOC)

Text S1 **Model formulation and INLA.**
(DOCX)

Text S2 **R code.**
(PDF)

Text S3 **Predicted cases by state and incidence maps under the assumption that missing values are zeros.**
(DOC)
