# Supplementary material for: Bayesian Geostatistical Modeling of Leishmaniasis Incidence in Brazil
Source: PLoS Negl Trop Dis. 2013 May 9;7(5):e2213. doi: 10.1371/journal.pntd.0002213 (PMC3649962; doi:10.1371/journal.pntd.0002213)
Supplement: Alternative Language Abstract S1 — Translation of the abstract into Portuguese by R. G. C. Scholte. (DOC) [file pntd.0002213.s001.doc]

## Modelagem geoestatística Bayesiana de Incidência da Leishmaniose no Brasil

***Antecedentes:*** A leishmaniose é endêmica em 98 países, com um número estimado de 350 milhões de pessoas em situação de risco e cerca de 2 milhões de casos por ano. O Brasil é um dos países mais afetados.
***Metodologia:*** Foram aplicados modelos geoestatísticos Bayesianos binomiais negativos para analisar os dados de incidência de leishmaniose tegumentar e visceral no Brasil, cobrindo um período de 10 anos (2001-2010). Particular ênfase foi colocada sobre os padrões espaciais e temporais. Os modelos foram ajustados usando aproximações aninhadas integradas Laplace para realizar rápidas inferências Bayesianas aproximadas. A seleção ​​Bayesiana de variáveis foi utilizada para determinar os preditores climáticos, ambientais e socioeconômicos mais importantes da leishmaniose cutânea e visceral.
***Achados principais:*** Para os dois tipos de leishmaniose, precipitação e variáveis socioeconômicos foram identificados como fatores de risco importantes. O número previsto de casos em 2010 foi de 30,189 (desvio padrão (DP): 7,676) para a leishmaniose cutânea e 4,889 (DP: 288) para a leishmaniose visceral. Nossos mapas de risco mostram o maior número de pessoas infectadas nos estados de Minas Gerais e do Pará para leishmaniose visceral e cutânea, respectivamente.
***Conclusões/Importância:*** Nossos mapas de alta resolução de incidência identificaram áreas prioritárias em que os esforços de controle de leishmaniose devem ser direcionados com o objetivo de reduzir a incidência da doença.
***Traduzido para o português:*** Ronaldo G. C. Scholte
