# Supplementary material for: Bayesian Geostatistical Modeling of Leishmaniasis Incidence in Brazil
Source: PLoS Negl Trop Dis. 2013 May 9;7(5):e2213. doi: 10.1371/journal.pntd.0002213 (PMC3649962; doi:10.1371/journal.pntd.0002213)
Supplement: Text S1 — Model formulation and INLA. (DOCX) [file pntd.0002213.s002.docx]

## Supporting Information Text 1

Here we present the model formulation, a brief description of the INLA approximation to estimate the marginal posterior distributions of the model parameters, and provide implementation details for the analysis of leishmaniasis data. The R code is given in supporting information text 2 (S2). Extensive theoretical explanations about INLA in a spatio-temporal setting have been presented elsewhere [55].

## Model formulation

Let $Y_{it}$ be the number of cases for municipality $i$ at year $t$. We assume that the $Y_{it}$’s are generated by a negative binomial distribution, i.e. $Y_{it}\sim NB(\mu_{it},k)$ with mean $\mu_{it}$ and dispersion parameter $k$. The linear predictor $\eta_{it}=\log(\mu_{it})=\log(P_{i})+X_{it}^{T}\beta+w_{i}+e_{t}$ includes an offset term for the population $P_{i}$, the vector $X_{it}^{T}$ of covariates, spatially and temporally structured random effects $w_{i}$and $e_{t}$, respectively. We consider that the vector of $w_{i}$ arises from a multivariate normal distribution $w\sim MVN(0,\Sigma)$with Matérn covariance function between locations $i,j$that is, , where $\sigma^{2}$ is the spatial process variance, $d_{ij}$is the distance between the centroids of $i,j,$ $\kappa$ is a scaling parameter, $v$ is a smoothing parameter fixed to 1 in our application and is the modified Bessel function of second kind and order $v$. The Matérn specification of the covariance matrix implies that the spatial range $r$, that is the distance at which spatial correlation becomes negligible (i.e., smaller than 10%) is $r=\frac{\sqrt{8}}{\kappa}$. We adopted a stationary autoregressive AR(1) process for $e_{t}$ such that, $e_{t}\sim N(\rho e_{t-1},\tau_{2}^{2})$for $t>1$ and $e_{1}\sim N(0,\tau_{1}^{2})$, where $\tau_{1}^{2}=\tau_{2}^{2}/(1-\rho^{2})$ and $\rho$ the auto-correlation parameter, constraint in the interval $(-1,1)$. We complete Bayesian model formulation by specifying prior distributions for the remaining parameters and five hyperparameters. In particular, we choose $\log$- gamma priors for $\tau_{2}^{-2}$, $\sigma^{-2}$, $r$and $k$ parametrized in the log scale, that is, $\log\left(\tau_{2}^{-2}\right)$, $\log\left(\sigma^{-2}\right)\sim\log Ga(1,0.0005)$, $\log(k)\sim\log Ga(1,1)$, $\log(r)\sim\log Ga(1,0.01)$. A normal prior distribution is used for $\rho$, re-parametrized in order to be defined in ℜ, that is $\log\left(\frac{1+\rho}{1-\rho}\right)\sim N(0,6.66)$. Normal priors $N(0,0.001)$ were also assigned for the regression coefficients and a vague normal one for the intercept.

## Bayesian inference using SPDE/INLA

Bayesian inference estimates the marginal (or full conditional) posterior distributions of the elements of the parameter vector , where $\theta$ is the vector of hyperparameters and $y$ are the data. Geostatistical models often rely on Markov chain Monte Carlo (MCMC) simulation to estimate . However computations involving the spatial covariance matrix are not feasible for large number of locations. Lindgren et al. (2011) [21] proposed the stochastic partial differential equations (SPDE) approach which represents the above Gaussian spatial process by a Gaussian Markov random field (GMRF). Hence $\Sigma$ is approximated by the covariance matrix $Q^{-1}$ of the GMRF, which provides directly the inverse of $Q$, overcoming a computationally intensive matrix operation. The spatial process representation is based on a partition of the study region into a set of non-intersecting triangles. Subsequently, INLA can be used for fast Bayesian inference. INLA approximates the above integral by $\hat{p}(\varphi_{j}|y)=\sum_{k} \hat{p}(\varphi_{j}|\theta_{l},y)\hat{p}(\theta_{l}|y)\omega_{l}$.$\hat{p}(\theta_{k}|y)$is calculated from the Laplace approximation of $p(\theta|y)$, that is , where is the Gaussian approximation of and is the mode of. $\hat{p}(\varphi_{j}|\theta_{l},y)$is also calculated from a Laplace approximation of $p(\varphi_{j}|\theta,y)$ and $\omega_{l}$ are weights associated with $\theta_{l}$. The prediction of the spatial random effect on a grid of locations is performed by projecting the triangular random effects on the grid and calculating a weighted sum of the values at the vertices. The weights are the barycentric coordinates of each grid point. Estimates of the total number of cases across states or the whole country can be obtained by summing pixel-level predictions. The INLA package does not provide directly variation measures for joint distributions and therefore, it cannot estimate the variance of the above quantities. However, it can estimate the variance of linear combinations of $\eta_{it}$ for a given time point $t$ (e.g. 2010). Using the Taylor expansion, the variance of the total predicted cases is given by: $Var\left(\sum_{i} \exp(\eta_{it})\right)\approx Var\left(\sum_{i} \exp(E(\eta_{it}))\eta_{it}\right)$ where the weights $\exp(E(\eta_{it}))$of the linear combination are the point predictions at pixel $i$. INLA can estimate the right part of the above equation in a second model fit which includes the prediction grid with missing values in the response. Additional linear combinations were defined to calculate the variance of the cases per state in a similar manner.

## INLA implementation

The data file contained standardized continuous predictors and the dummy (0/1) variables of the categorical ones. We assigned a missing value to the response of a randomly selected set of 20% of the data (test data). The response was predicted for these points and used to calculate cross-validatory measures.

The R package "maps" [56] was used to define the boundaries of our region that was triangulated. The inla.mesh.create.helper(), inla.spde2.matern() functions, of the INLA package, were applied to construct the domain (mesh) and define the covariance function of the spatial process. The inla() was called to perform approximate Bayesian inference and obtain summaries for the coefficients and the hyper-parameters. The grid for prediction was constructed with the inla.mesh.projector(). inla.mesh.project() projected the mean of the latent spatial effect on the grid. Using ArcMap 10.0, covariate values and the population data were extracted at the grid points which are later read in R. The mean of the linear predictor was calculated and summarized over the states to approximate the predicted cases. Finally, a second inla() call enabled the estimation of the variance of the cases aggregated over the whole country and states.

## Additional References

55. Cameletti M, Lindgren F, Simpson D, Rue H (2012) Spatio-temporal modeling of particulate matter concentration through the SPDE approach. Adv Stat Anal. 10.1007/s10182-012-0196-3.

56. Becker RA, Wilks AR, Brownrigg R, Minka TP (2012) Maps: draw geographical maps R package version 2.2-6 <http://CRAN.R-project.org/package=maps>.
