# Supplementary material for: Bayesian Geostatistical Modeling of Leishmaniasis Incidence in Brazil
Source: PLoS Negl Trop Dis. 2013 May 9;7(5):e2213. doi: 10.1371/journal.pntd.0002213 (PMC3649962; doi:10.1371/journal.pntd.0002213)
Supplement: Text S2 — R code. (PDF) [file pntd.0002213.s003.pdf]

Supporting Information Text 2

R code

```
library(INLA)
library(maps)
library(foreign)

# read data
cl.data <- read.dbf("cutaneous_data.dbf")
# define subset for cross validation (test data)
cl.data$new.cases <- cl.data$cases
id.cross <- sample(c(1:nrow(cl.data)),ceiling(nrow(cl.data)*0.8))
cl.data$new.cases[-id.cross] <- NA
link <- rep(NA,nrow(cl.data))
link[which(is.na(cl.data$new.cases))] <- 1

# region boundaries
boundaries <- map("world","brazil")
# domain construction
cl.coords <- cbind(cl.data$x,cl.data$y)
mesh <- inla.mesh.create.helper(points=as.matrix(cl.coords),
                    points.domain=cbind(boundaries$x,boundaries$y),
                    offset=c(0.1,1),
                    max.edge=c(1,50),
                    min.angle=c(26,21),
                    cutoff=0,plot.delay=NULL)
spde <- inla.spde2.matern(mesh=mesh,alpha=2)
mesh$n #8098
field <- mesh$idx$loc

# model fit
total.pop <- cl.data$total_pop
#
formula <- new.cases ~ bioclim22+bioclim23+bioclim24+
                    bioclim8+bioclim12+bioclim15+
                    bioclim182+bioclim183+bioclim184+
                    bras0_112+bras0_113+bras0_114+
                    bras0_12+bras0_14+
                    evi_05_092+evi_05_093+evi_05_094+
                    feminino+
                    hii2+hii3+hii4+
                    lst_day_05+
                    trend+
                    f(field,model=spde)+f(year,model="ar1")
#
```

```
cl.result <- inla(formula,data=cl.data,family="nbinomial",E=total.pop,
                  control.compute=list(dic=T,cpo=T),
                  control.predictor=list(compute=TRUE,
                                         quantiles=c(0.025,0.5,0.975),
                                         link=link),
                  verbose=T)

# to obtain summaries of the spatial parameters
result.field <- inla.spde.result(cl.result,"field",spde,do.transform=T)
# eg for the range
inla.qmarginal(c(0.025,0.5,0.975),result.field$marginals.range.nominal[[1]])
# incidence rate ratio by transforming the marginals
# eg for trend (period after 2005)
irrs <- inla.tmarginal(function(x)exp(x),result$marginals.fixed$trend)
inla.qmarginal(c(0.025,0.5,0.975),irrs)
inla.emarginal(function(x)x,irrs)

# cross-validatory measures
fitted.values <- cl.result$summary.fitted.values[is.na(link)==F,1]
observed.values <- cl.data$cases[is.na(link)==F]
rmse <- sqrt(mean((observed.values-fitted.values)^2))
fit0025 <- cl.result$summary.fitted.values[is.na(link)==F,"0.025quant"])
fit0975 <- cl.result$summary.fitted.values[is.na(link)==F,"0.975quant"])
coverage <- mean((observed.values>=fit0025)&(observ.val<=fit0975))

# define a grid and project the values of the latent field
prog_grid <- inla.mesh.projector(mesh,xlim=c(-73.93801,-34.91713),
                  ylim=c(-33.66801,5.13689),
                  dims=c(543,540))
spatial.mean <- matrix(cl.result $summary.random$field[,"mean"],mesh$n,T)
spatial.mean.grid <- inla.mesh.project(prog_grid,spatial.mean[,1])
# extract the points with their random effect value
temp <- levelplot(row.values=prog_grid$x,column.values=prog_grid$y,
                  x=spatial.mean.grid,xlim=c(-77,-30),ylim=c(-35,10))
to.arcmap <- cbind(temp$panel.args.common$x,
                  temp$panel.args.common$y,
                  temp$panel.args.common$z)
colnames(to.arcmap) <- c("x","y","latent")
write.dbf(to.arcmap,"cl_latent.dbf")
# in ArcMap 10.0 "cut" the grid and delete points outside Brazil,
# extract the closest covariate values and population,
# and assign the state to which each point belongs
cl.closest <- read.dbf("cl_closest.dbf")

# calculate the linear predictor
coefs <- (cl.result$summary.fixed[,1])
names(coefs)[1] <- "constant"
```

```
cl.closest$constant <- 1
cl.lp <- as.matrix(cl.closest[,names(coefs)])%*%coefs +
                    cl.closest$latent+
                    cl.result$summary.random$year[10,2] #for 2010
#
state <- cl.closest$NAME_1
nstates <- length(unique(state))
states <- unique(state)
states <- states[order(states)]
# calculate weights $\exp(E(\eta_{it}))$
cl.lp <- as.numeric(cl.lp)
cl.weights <- exp(cl.lp)*cl.closest$population
cl.in.states <- aggregate(cl.weights,FUN=sum,by=list(state)) # predicted cases by state

# linear combinations for variance calculation
country <- inla.make.lincomb(Predictor=c(rep(NA,nrow(cl.data)),cl.weights))
lc.list <- as.list(rep(NA,nstates))
for(i in 1:nstates){
                    temp <- rep(NA,nrow(cl.closest))
                    temp[state==states[i]] <- weights[state==states[i]]
                    temp <- c(rep(NA,nrow(cl.data)),temp)
                    lc.list[i] <- inla.make.lincomb(Predictor=temp)
}
# new mesh and spde with all coords
cl.predict.coords <- cbind(cl.closest$x,cl.closest$y)
cl.bind.coords <- rbind(cl.coords,cl.predict.coords)
mesh <- inla.mesh.create.helper(points=as.matrix(cl.bind.coords),
                    points.domain=cbind(boundaries$x,boundaries$y),
                    offset=c(0.1,1),
                    max.edge=c(1,50),
                    min.angle=c(26,21),
                    cutoff=0)
mesh$n #160769
field <- mesh$idx$loc
spde <- inla.spde2.matern(mesh=mesh,alpha=2)
#
total.pop.binded <- c(total.pop,cl.closest$population)
cl.closest$cases <- NA
cl.closest$year <- 10
cl.binded <- rbind(cl.data[,c("cases",names(coefs)[-1])],
                    cl.closest[,c("cases",names(coefs)[-1])])
link <- rep(NA,nrow(cl.binded))
link[which(is.na(cl.binded$cases))] <- 1
#
cl.result2 <- inla(formula,data=cl.binded,family="nbinomial",E=total.pop.binded,
                    lincomb=c(lc,lc.list),
                    control.compute=list(dic=T,cpo=T),
```

```
                    control.predictor=list(compute=TRUE,
                                            quantiles=c(0.025,0.5,0.975),
                                            link=link),
                    verbose=T,inla.call="remote",num.threads=12,keep=T)
states.sd <- cbind(states,round(cl.result2$summary.lincomb.derived[1:nstates,"sd"],2))
```
