# Supplementary material for: Bayesian Geostatistical Modeling of Leishmaniasis Incidence in Brazil
Source: PLoS Negl Trop Dis. 2013 May 9;7(5):e2213. doi: 10.1371/journal.pntd.0002213 (PMC3649962; doi:10.1371/journal.pntd.0002213)

## Supporting Information Text 3

This supplementary material contains results from Bayesian Modeling of leishmaniasis incidence, under the assumption that missing data are zero cases.

**Table 6**: Country and state predicted cases of cutaneous leishmaniasis (CL) and visceral leishmaniasis (VL) in Brazil in 2010.

| State | CL cases | VL cases |
| --- | --- | --- |
| Acre | 1,791.0 | 0.1 |
| Alagoas | 85.0 | 96.5 |
| Amapá | 462.6 | 0.5 |
| Amazonas | 2,810.5 | 2.2 |
| Bahia | 2,237.6 | 410.0 |
| Ceará | 1,152.7 | 505.1 |
| Distrito Federal | 81.4 | 12.6 |
| Espírito Santo | 232.0 | 3.6 |
| Goiás | 478.9 | 35.1 |
| Maranhão | 3,016.7 | 401.6 |
| Mato Grosso | 3,440.4 | 27.5 |
| Mato Grosso do Sul | 192.7 | 203.6 |
| Minas Gerais | 1,619.6 | 417.9 |
| Pará | 4,227.8 | 484.3 |
| Paraíba | 63.9 | 40.0 |
| Paraná | 645.6 | 2.9 |
| Pernambuco | 605.2 | 143.9 |
| Piauí | 198.3 | 263.4 |
| Rio de Janeiro | 298.2 | 4.5 |
| Rio Grande do Norte | 11.7 | 87.1 |
| Rio Grande do Sul | 10.8 | 146.2 |
| Rondônia | 1,743.8 | 0.9 |
| Roraima | 249.9 | 7.6 |
| Santa Catarina | 61.2 | 48.9 |
| São Paulo | 796.2 | 155.7 |
| Sergipe | 27.9 | 58.9 |
| Tocantins | 743.5 | 256.6 |
| Total | 27,285.0 | 3,817.4 |

**Figure 6. Geostatistical model-based predicted incidence rates per 10,000 for cutaneous leishmaniasis in Brazil in 2010.**


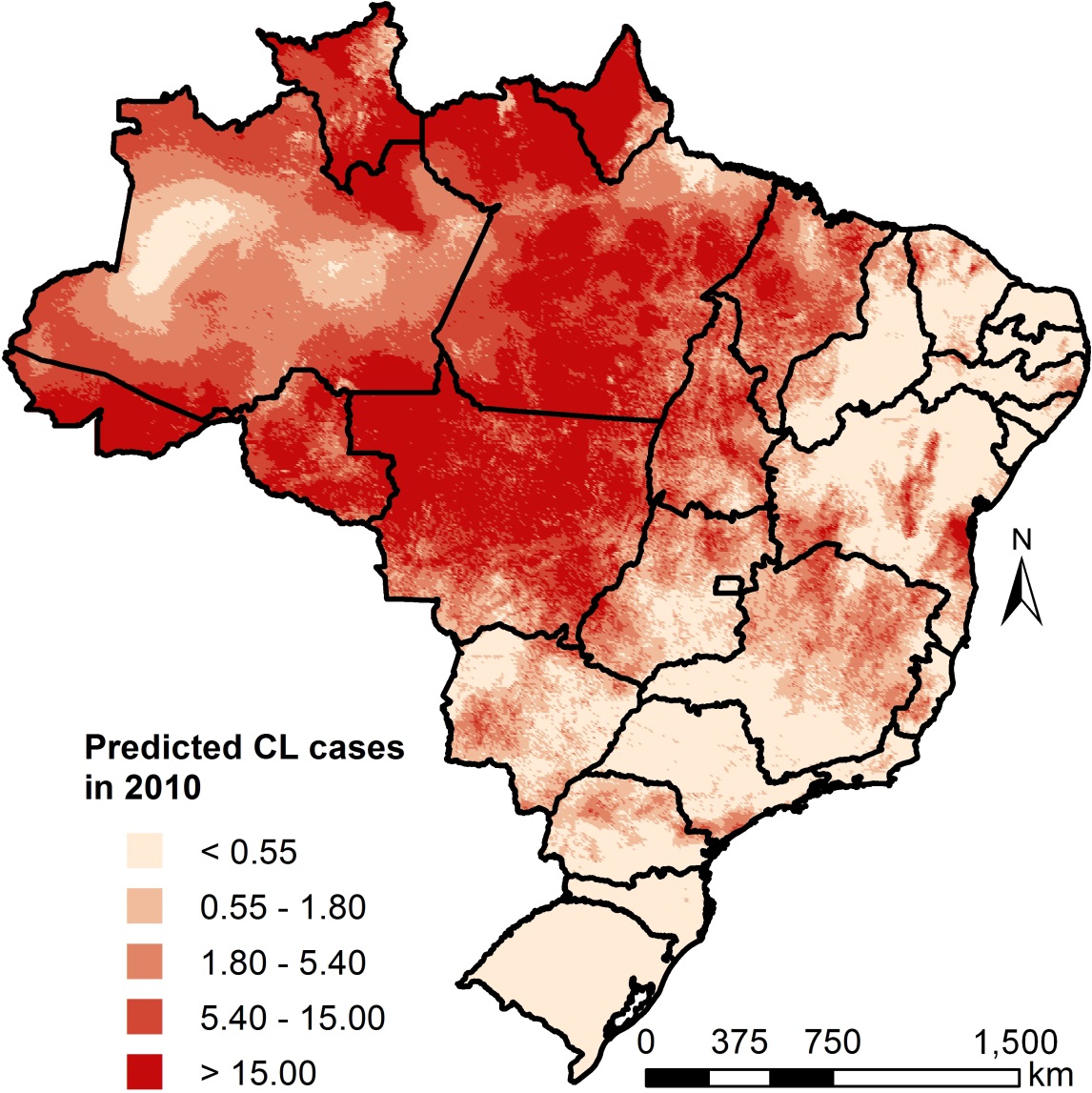


**Figure 7. Geostatistical model-based predicted incidence rates per 10,000 for visceral leishmaniasis in Brazil in 2010.**


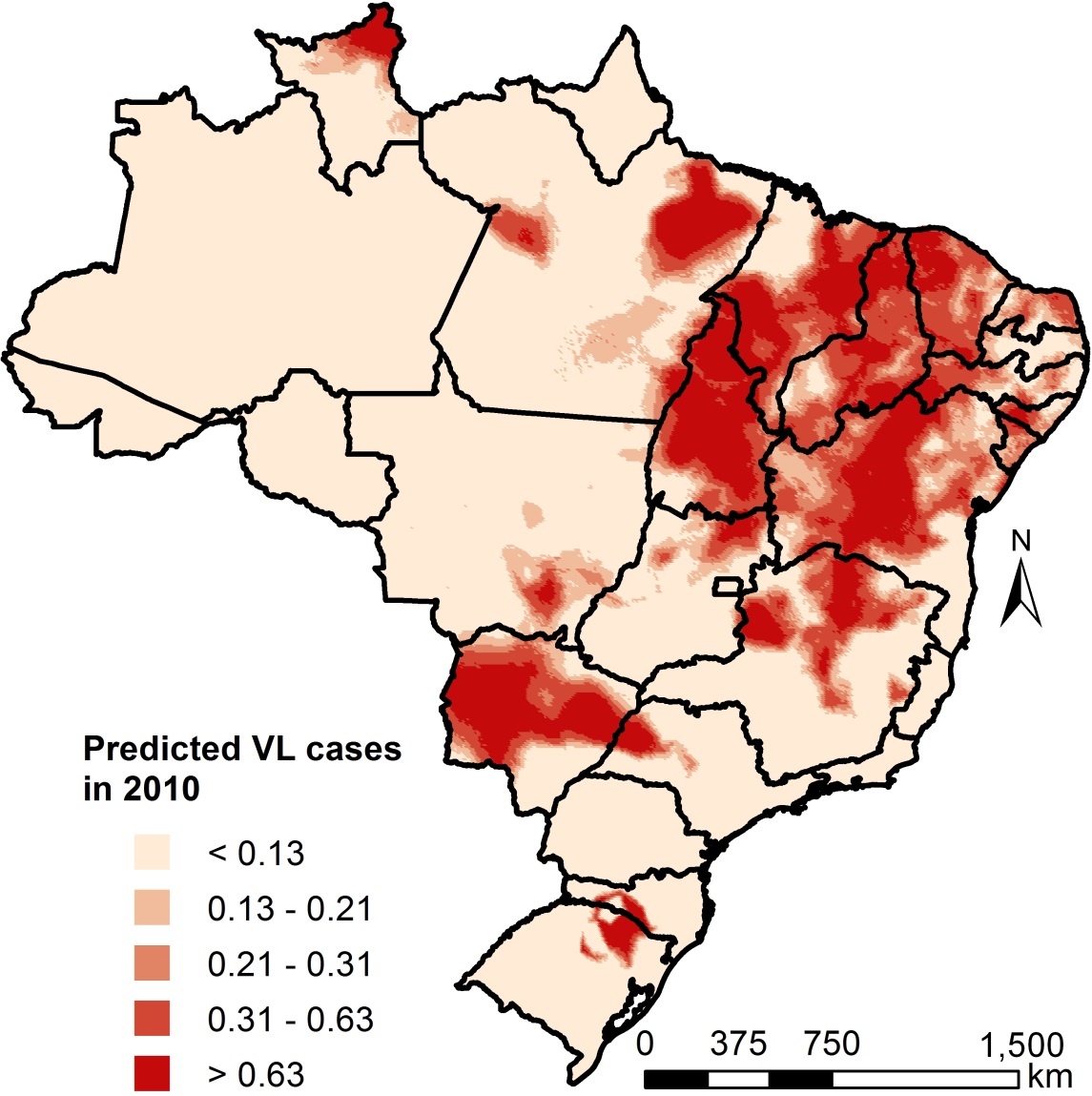

Supplement: Text S3 — Predicted cases by state and incidence maps under the assumption that missing values are zeros. (DOC) [file pntd.0002213.s004.doc]
